# Epidemiological profile of dog attacks to patients under 14 years old assisted at the pediatric referral emergency unit of a tertiary hospital in Campinas, Brazil

**DOI:** 10.3389/fped.2022.963803

**Published:** 2022-08-03

**Authors:** Michelle Marchi Medeiros, Fernando Augusto Lima Marson, Leonardo Souza Marques, Andressa Oliveira Peixoto, Andrea de Melo Alexandre Fraga

**Affiliations:** ^1^Department of Pediatrics, University of Campinas, Campinas, Brazil; ^2^Laboratory of Medical and Human Genetics, São Francisco University, Braganca Paulista, Brazil

**Keywords:** *Canis lupus familiaris*, dog bite, emergency, pediatrics, trauma

## Abstract

**Introduction:**

Accidents involving dog attacks are very common, which makes this type of accident a global public health issue. The estimates point to 20% of the victims of such accidents seeking care in health units, and half of them being children. In addition to acute injuries, dog attacks might result in fractures, infections, scars, and psychological traumas. This study aimed to describe the epidemiological profile of dog attacks to children under 14 years old assisted in a pediatric emergency service in Brazil.

**Methods:**

The database of the Information and Toxicological Assistance Center of Campinas was surveyed to identify cases of children under 14 years old assisted after a dog attack in a 9-years period. Demographic data, number and type of lesions, type of exposure, part of the body affected, dog origin and condition, and the accident location and cause were analyzed. The data were presented in a descriptive way, and the age groups were classified as follows: 0–3 years old, 4–6 years old, and 7–14 years old. The different age groups were compared one to another regarding the markers evaluated using the chi-square test and the Fisher’s exact test. A 0.05 alpha was adopted in all analyses.

**Results:**

The number of children assisted in the study period totaled 1,012. The 7–14-year-old group was the most affected (*n* = 498; 49.2%), male patients were also majority (*n* = 660; 65.2%). Most injuries were found on the head/neck area (*n* = 378; 37.4%). However, the older the patients were, the higher the frequency of lesions on upper and lower limbs was, as well as attacks occurred in external environments, thus involving animals that could not be observed. A significant increase in accidents with provoked causes was observed in younger patients.

**Conclusion:**

Accidents involving dog attacks are more likely to happen among boys. Younger children run higher risks of becoming victims of these accidents inside homes, being attacked by pets, and showing a greater incidence of head and neck lesions. Older children present more injuries on their limbs, which are caused by dogs that cannot be observed.

## Introduction

External causes, which include several types of accidents and violence, remain relevant factors in child mortality and permanent disability rates worldwide. In Brazil, around 3,300 children from 1 to 14 years old die, while around 112,000 are hospitalized due to accidents and violence every year (1). Despite the beneficial effects of animal contact with human beings for reducing loneliness and improving wellbeing (2), for example, accidents related to dog bites (*Canis lupus familiaris*) are common in Brazil and abroad, making this kind of accident a global public health issue (3). There are around 139 million pets in Brazil, and among these, dogs are the most common, representing 40% of domestic animals (4).

American data has shown that around 4.5 million dog bites occur every year in the United States of America, and half of them involve attacks to children (5, 6). The chances of a child experiencing a dog attack during their lives is estimated to be around 50% (7, 8). Approximately 800 thousand cases of dog bites require some type of medical care, generating costs of up to two billion dollars a year to health services (3, 5, 7, 9).

In American pediatric emergency units, dog bites are among the top ten causes of childcare from 1 to 9 years old, and in a great part of populational studies on these accidents around the world, the pediatric age group is the most affected (9–12). Most studies on dog attacks in the child population are carried out with data collected from emergency assistance or hospital admission for treatment, which suggests that the actual incidence of dog bites in children might be even higher than that reported since the estimates point to only 20% of the victims being taken to health services for medical care (3).

Several studies have shown that lesions caused by dog bites are more common in specific demographic groups. Male children in school age are the most affected group (3, 5, 12–15). Children under 5 years old are usually bitten on the head and neck, while the older ones have their upper and lower limbs mostly bitten (3, 5–7, 9, 10, 14, 16, 17). Most accidents with babies and pre-school children occur inside their homes, and with dogs that the child is familiar with, while adolescents are usually involved in accidents with dogs, they do not know (3, 5, 10, 12–14, 16).

In addition to acute injuries, these attacks might cause fractures, infections, scars, and psychological traumas (14, 15, 17). Around 50% of the victims need some type of repair, many requiring sedation or anesthesia for procedures from sutures to surgeries such as debridement and grafts, in addition to the use of antibiotics and hospital care (5, 9, 12, 14, 15, 18, 19). Even with suitable treatment of the injuries, some physical and psychological consequences might remain. There are reports in the literature that 70% of the parents of children victims of dog bites noticed behavioral alterations in their children in the long term after the accident (5, 13). Some accidents might be serious and result in death, while accidents, even if not so serious, might result in irreversible consequences (20, 21). There is some evidence that 56% of casualties related to dog bites occur with children under 16 years old (5, 9).

Some dog breeds are more commonly involved in reports of attacks to human beings. Dogs of the breeds Pit Bull, Rottweiler, and German Shepherd, probably due to their specific characteristics such as strength and jaw shape, usually present more severe bites that require medical care and are frequently cited in studies (5, 11, 12, 14, 16, 17, 19, 20, 22, 23). Curiously, dogs of the breeds Labrador, Golden Retriever, Border Collie, Jack Russel, and Shih-Tzu, despite being considered good pets for families with children, also present a high incidence of attacks with bites (5, 11, 14, 16, 17, 22), but these injuries are usually less serious.

Most children victims of dog bites are attacked by their own dogs or dogs they are used to (3, 5, 12, 14, 24), which shows that regardless of the breed, child/dog interaction is a relevant factor in the occurrence of an attack. Even apparently positive activities might not be completely safe (for example, the child trying to play, hug, or feed the animal). It is believed that the child’s familiarity with the dog provokes a feeling of safety, allowing a longer time of unsupervised interaction between the child and the animal (5). In such context, small educational interventions might help to develop a safe behavior when being close to the dog and improve the child’s ability to interpret signs of danger of a possible attack (10, 11).

In Brazil, there are few studies on the epidemiology of dog attacks in the pediatric age group. Data that characterize the epidemiology and the circumstances involved in the dog attacks are important to understand risk factors and instruct the development of strategies to prevent these accidents. Thus, this study aimed to describe the epidemiological profile of dog attacks in patients under 14 years old in a Pediatric referral emergency unit in a tertiary hospital in Campinas, Brazil.

## Materials and methods

A retrospective study was carried out to describe the epidemiology of accidents involving dogs and children in the region of Campinas—SP, Brazil. All medical care given to children victims of dog attacks at the Pediatric referral emergency of the *Hospital de Clinicas* at the University of Campinas from January 2010 to December 2019 were included in the study. This hospital is one of the largest university hospitals in Brazil and a national referral center of tertiary services. Its highly complex assistance capillarity is a reference in the municipality of Campinas and its microregion that comprises 62 municipalities of the VII and XIV regional health departments, assisting around 6.5 million inhabitants.

The information included in this study was collected from the medical records found in the *Centro de Informação e Assistência Toxicológica de Campinas*—CIATox (Information and Toxicological Assistance Center of Campinas). CIATox is part of the Faculty of Medical Sciences of the University of Campinas and is one of the support services of the *Hospital de Clinicas*, being responsible for the application of anti-rabies immunobiological agents when prescribed in the prophylaxis of human rabies.

Children from 0 to 14 years old who received medical care at the pediatric referral emergency unit after a dog attack were included in the study. The following variables were analyzed: (i) sex (male and female); (ii) age group (classified as: 0–3 years old, 4–6 years old, and 7–14 years old); (iii) number of lesions (single or multiple); (iv) type of lesion (superficial, deep, or tearing); (v) type of exposure (scratch, lick, and bite); (vi) part of the body injured (head/neck including face, trunk, upper limbs including hands, lower limbs including feet, and mucosa including mouth and eyes); (vii) dog’s origin [pet (domiciled dog), semi-domiciled, and non-domiciled]; (viii) animal’s condition (disappeared, ill, dead, and healthy); (ix) accident location (children’s own house, or external environment—e.g., street, family members’ house, neighbors, acquaintances, and school); (x) region where the accident occurred (rural or urban); (xi) children’s origin (rural or urban); and (xii) cause of the dog attack (accidental or provoked).

We considered domiciled animals those that are completely dependent on their owners, who only leave their homes accompanied and restrained using collar and harness, are vaccinated, and submitted to periodical clinical control. Semi-domiciled are those that depend on their owners but can be found outside their homes, unaccompanied, and for undetermined periods. However, they also are vaccinated and receive some care. Finally, the non-domiciled are independent dogs that live free on the streets, small rural properties, or farms, and do not receive any kind of care. As for the classification of the type of attack, we considered it accidental when the child was not interacting with the dog when attacked, while it was considered provoked when the child was involved with the dog at the time of the accident, either playing, patting, or any other kind of direct interaction.

The statistical analysis was aided by the software Statistical Package for the Social Sciences (IBM SPSS Statistics for Macintosh, Version 27.0). The descriptive data were presented as absolute frequency (*n*) and relative frequency (%). The inference analysis was carried out using the chi-square test and Fisher’s exact test. All analyses considered a 0.05 alpha error. No technique was employed to the missing data input since the data loss was below 5%, except for the animal breed that presented a high occurrence of missing data.

## Results

**FIGURE 1:**
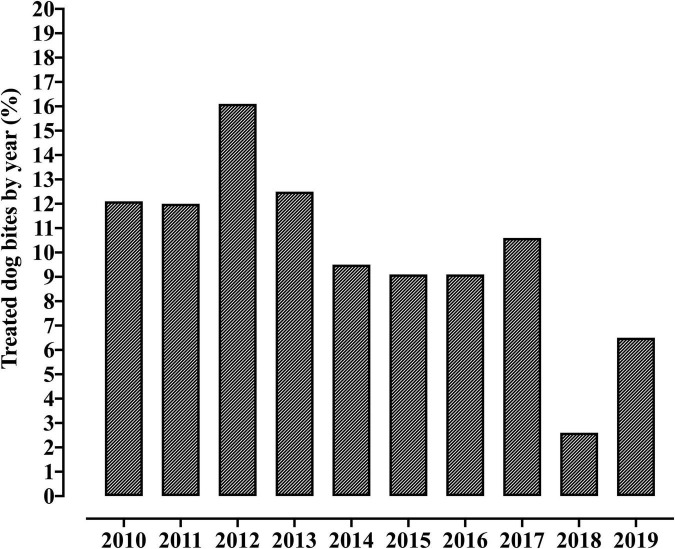
Distribution of cases of dog bites in individuals under 14 years old assisted at the Pediatric referral emergency unit of a tertiary hospital in Campinas, Brazil, according to the year of inclusion.

**FIGURE 2:**
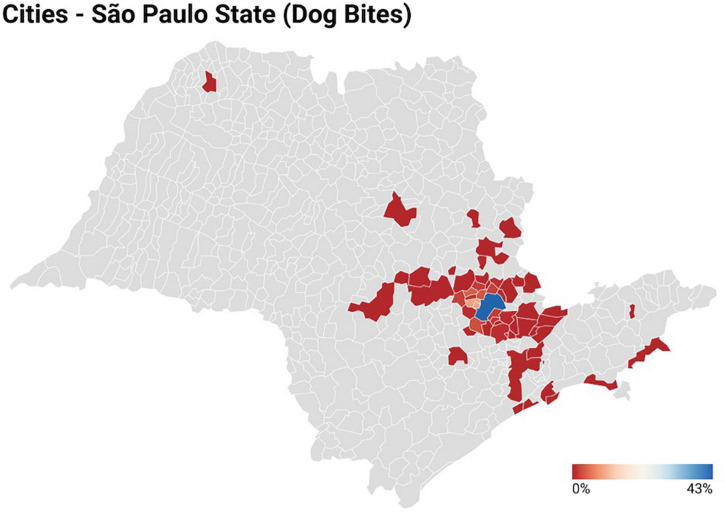
Distribution of cases of dog bites in individuals under 14 years old assisted at the Pediatric referral emergency unit of a tertiary hospital in Campinas, Brazil, according to the city where the accident occurred. Four cases occurred in Minas Gerais state, one case in Paraná state, and 25 other cases were not included because the municipality where the accident occurred was not reported.

In the period from January 2010 and December 2019, 1,012 children that were victims of dog attacks were assisted at the referral center. In our hospital, we assisted near of 12,800 children in the emergency unit by year, in such a scenario, we assisted ∼0.9% [1,012/(12,800 × 9)] of the cases due to Dog bites. Among these, only 135 (13.3%) patients had previous assistance by phone (Table 1). The year with the highest number of cases reported was 2012 (*n* = 163; 16.1%), and the average of care provided was 112.4 cases a year. In 2018, the service was restructured, and the register of cases changed, which resulted in greater difficulty to access the medical records. In 2019, a reduction in the number of cases was observed (*n* = 66; 6.5%) when compared to 2010–2017 (Figure 1 and Supplementary Table 1), possibly due to some bias in the data collection. The distribution of cases according to the municipality where they occurred is presented below (Figure 2 and Supplementary Table 2).

**TABLE 1:** Epidemiological profile of individuals under 14 years assisted at the pediatric referral emergency unit of a tertiary hospital in Campinas, Brazil, after dog attacks.

Marker	Groups	*N* (%)
Type of care	At the unit	877 (86.7%)
	Telephone + at the unit	135 (13.3%)
Sex	Female	352 (34.8%)
	Male	660 (65.2%)
Age (years)	0–3 years old	246 (24.3%)
	4–6 years old	268 (26.5%)
	7–14 years old	498 (49.2%)
Victim’s origin	Rural	51 (5.0%)
	Urban	958 (94.7%)
	Unknown	3 (0.3%)
Accident region	Rural	85 (8.4%)
	Urban	902 (89.1%)
	Unknown	25 (2.5%)
Accident location	Home	317 (31.3%)
	Street	690 (68.2%)
	Unknown	5 (0.5%)
Dog’s origin	Domiciled	450 (44.5%)
	Non-domiciled	394 (38.9%)
	Semi-domiciled	143 (14.1%)
	Unknown	25 (2.5%)
Animal’s condition	Disappeared	395 (39.0%)
	Ill	1 (0.1%)
	Dead	50 (4.9%)
	Healthy	560 (55.3%)
	Unknown	6 (0.6%)
Cause of the accident	Accidental	657 (64.9%)
	Provoked	309 (30.5%)
	Unknown	46 (4.5%)

Data presented as the number of individuals (N) and percentage (%).

The dog breed was only informed in 49.4% (*n* = 500) of the medical records. The breeds found totaled 26, and the mongrel (mixed-breed) dogs were the most common (*n* = 366; 36.2%), followed by Pit Bulls (*n* = 36; 3.6%), German Shepherds (*n* = 14; 1.4%), and Poodles (*n* = 13; 1.3%) (Supplementary Table 3).

Boys were more often attacked (*n* = 660; 65.2%) than girls (*n* = 352; 34.8%). Lesions were mostly found in the 7–14 years old group, representing 498 (49.2%) patients, followed by the 4–6 years old group, with 268 (26.5%) children, and the 0–3 years old group with 246 (24.3%) cases (Table 1). Most of the children assisted lived in the urban region (*n* = 958; 94.7%), and most of the accidents occurred in the urban region too (*n* = 902; 89.1%).

The accidents were mainly caused by healthy (*n* = 560; 55.3%) and domiciled (*n* = 450; 44.5%) dogs. In 394 (38.9%) cases, the dogs were non-domiciled, in 143 cases (14.1%), the animals were semi-domiciled, and a small percentage of records did not report the origin of the dogs (*n* = 25; 2.5%).

When analyzing the origin of the dogs involved in the accidents in relation to the different age groups, we could observe that in the 7–14 years old group, domiciled dogs were the most prevalent (*n* = 237; 49.2%). In 175 (36.3%) cases, this age group was attacked by domiciled dogs, and in 70 (14.5%) cases, the dogs were semi-domiciled. In the 0–3 years old and 4–6 years old groups, accidents involving domiciled dogs were the most common, corresponding to 132 (54.8%) and 143 (54.2%) cases, respectively. Non-domiciled dogs were involved in 70 (29%) accidents in the 0–3 years old group and in 87 (33%) accidents in the 4–6 years old groups. In 39 (16.2%) cases involving 0–3 years old children, and in 34 (12.9%) cases in the 4–6 years old group, semi-domiciled dogs were responsible for the attack (Table 2). We could also observe that when the children’s age increased, the number of accidents involving non-domiciled dogs also increased, while when younger children were investigated, the number of accidents involving domiciled dogs was higher (*P* < 0.001).

**TABLE 2:** Association of the epidemiological profile of individuals under 14 years old assisted at the pediatric referral emergency unit of a tertiary hospital in Campinas, Brazil, after dog attacks in the age groups investigated in the study.

Marker	Age				*P*
Sex	0–3 years old	4–6 years old	7–14 years old	Total	
Female	98 (39.8%)	93 (34.7%)	161 (32.3%)	352 (34.8%)	0.129
Male	148 (60.2%)	175 (65.3%)	337 (67.7%)	660 (65.2%)	
**Victim’s origin**					
Rural	12 (4.9%)	15 (5.6%)	24 (4.8%)	51 (5.1%)	0.894
Urban	234 (95.1%)	253 (94.4%)	471 (95.2%)	958 (94.9%)	
**Accident region**					
Rural	26 (11.0%)	25 (9.5%)	34 (7.0%)	85 (8.6%)	0.171
Urban	211 (89.0%)	239 (90.5%)	452 (93.0%)	902 (91.4%)	
**Accident location**					
Home	107 (44.0%)	97 (36.2%)	113 (22.8%)	317 (31.5%)	<0.001
Street	136 (56.0%)	171 (63.8%)	383 (77.2%)	690 (68.5%)	
**Dog’s origin**					
Domiciled	132 (54.8%)	143 (54.2%)	175 (36.3%)	450 (45.6%)	<0.001
Non-domiciled	70 (29.0%)	87 (33.0%)	237 (49.2%)	394 (39.9%)	
Semi-domiciled	39 (16.2%)	34 (12.9%	70 (14.5%)	143 (14.5%)	
**Animal’s condition**					
Disappeared	73 (29.8%)	87 (32.6%)	235 (47.6%)	395 (39.3%)	<0.001
Ill	0 (0.0%)	0 (0.0%)	1 (0.2%)	1 (0.1%)	
Dead	10 (4.1%)	9 (3.4%)	31 (6.3%)	50 (5.0%)	
Healthy	162 (66.1%)	171 (64.0%)	227 (46.0%)	560 (55.7%)	
**Cause of the accident**					
Accidental	130 (56.5%)	168 (66.1%)	359 (74.5%)	657 (68.0%)	<0.001
Provoked	100 (43.5%)	86 (33.9%)	123 (25.5%)	309 (32.0%)	
**Upper limb lesion**					
Yes	76 (30.9%	86 (32.1%)	182 (36.5%)	344 (34.0%)	0.231
No	170 (69.1%)	182 (67.9%)	316 (63.5%)	668 (66.0%)	
**Trunk lesion**					
Yes	13 (5.3%)	26 (9.7%)	56 (11.2%)	95 (9.4%)	0.031
No	233 (94.7%)	242 (90.3%)	442 (88.8%)	917 (90.6%)	
**Head and neck lesion**					
Yes	151 (61.4%)	127 (47.4%)	100 (20.1%)	378 (37.4%)	<0.001
No	95 (38.6%)	141 (52.6%)	398 (79.9%)	634 (62.6%)	
**Lower limb lesion**					
Yes	21 (8.5%)	49 (18.3%)	212 (42.6%)	282 (27.9%)	<0.001
No	225 (91.5%)	219 (81.7%)	286 (57.4%)	730 (72.1%)	
**Mucosa lesion**					
Yes	2 (0.8%)	7 (2.6%)	5 (1.0%)	14 (1.4%)	0.130
No	244 (99.2%)	261 (97.4%)	493 (99.0%)	998 (98.6%)	
**Number of lesions**					
Multiple	157 (65.1%)	157 (59.2%)	310 (63.0%)	624 (62.5%)	0.373
Single	84 (34.9%)	108 (40.8%)	182 (37.0%)	374 (37.5%)	

Data presented as the number of individuals (N) and percentage (%).

The statistical analysis was aided by the software Statistical Package for the Social Sciences (IBM SPSS Statistics for Macintosh, Version 27.0). The inference analysis was carried out using the chi-square test and Fisher’s exact test. All analyses considered a 0.05 alpha error.

Regarding the animals’ conditions, 395 (39%) were not found (disappeared), 50 (4.9%) animals were killed or died within 10 days after the date of the accident, and only one (0.1%) animal was ill, but the illness was not described in the medical records. Only six cases did not present the animal’s condition (Table 1). We verified that the frequency of attacks by animals that could not be observed increased when the children’s age increased (*P* < 0.001).

Most accidents occurred in external environments (*n* = 690; 68.2%). In 317 (31.5%) cases, the attacks occurred at the children’s homes, and in five (0.5%) cases, the accident location was not informed (Table 1). As for the age group, the cases were distributed as follows: (0–3 years old group) 136 (56%) cases occurred in an external environment, while 107 (44%) cases occurred at home; (4–6 years old group) 171 (63.8%) cases occurred in an external environment, while 97 (36.2%) cases occurred at home; and (7–14 years old group) 383 (77.2%) cases occurred in an external environment, while 113 (22.8%) cases occurred at home (Table 2). We also verified that the older the patients were, the higher the number of attacks occurred in external environments was (*P* < 0.001).

Most attacks had accidental causes (*n* = 657; 64.9%), and when each age group was investigated in isolation, the accidental cause was the most frequent (Tables 1, 2). However, a significant increase in the provoked cause was observed when the children’s age was reduced (*P* < 0.001).

Lesions on the head and neck were the most prevalent (*n* = 378; 37.4%), followed by lesions on upper limbs (*n* = 344; 34%), lower limbs (*n* = 282; 27.9%), trunk (*n* = 95; 9.4%), and mucosa (*n* = 14; 1.4%) (Table 3). Head and neck injuries were mostly found in younger children (*n* = 151; 61.4%). Regarding the lesions found on upper and lower limbs, they were mostly described in the 7–14 years old group (*n* = 182; 36.5% and *n* = 212; 42.6%, respectively). The 4–6 years old group presented head and neck injuries in 127 (47.4%) cases, while the 7–14 years old group presented 100 (20.1%) cases. Lesions on upper limbs appeared in 76 (30.9%) children aged 0–3 years old group and in 86 (32.1%) children in the 4–6 years old group, while lower limb injuries were reported in 21 (8.5%) children in the 0–3 years old group, and in 49 (18.3%) children in the 4–6 years old group. We observed that when the patient’s age increased, the frequency of head and neck lesions decreased, but lesions on upper and lower limbs increased (*P* < 0.001) (Table 2).

**TABLE 3:** Descriptive profile of the type of lesion and exposure of individuals under 14 years old assisted at the pediatric referral emergency unit of a tertiary hospital in Campinas, Brazil, after dog attacks.

Part of the body/Type of lesion	Groups	*N* (%)
Upper limb lesion	Yes	344 (34.0%)
	No	668 (66.0%)
Type of upper limb lesion	Tearing	7 (2.0%)
	Deep	300 (87.2%)
	Superficial	30 (8.7%)
	Unknown	7 (2.0%)
Exposure on upper limb	Scratch	13 (3.8%)
	Lick	4 (1.2%)
	Bite	320 (93.0%)
	Unknown	7 (2.0%)
Trunk lesion	Yes	95 (9.4%)
	No	917 (90.6%)
Type of trunk lesion	Deep	83 (87.4%)
	Superficial	12 (12.6%)
Exposure on trunk	Scratch	9 (9.5%)
	Lick	1 (1.1%)
	Bite	84 (88.4%)
	Unknown	1 (1.1%)
Head/neck lesion	Yes	378 (37.4%)
	No	634 (62.9%)
Type of head/neck lesion	Tearing	29 (7.7%)
	Deep	320 (84.7%)
	Superficial	26 (6.9%)
	Unknown	3 (0.8%)
Exposure on head/neck	Scratch	19 (5%)
	Bite	359 (95%)
Lower limb lesion	Yes	282 (27.9%)
	No	730 (72.1%)
Type of lower limb lesion	Tearing	14 (5.0%)
	Deep	240 (85.1%)
	Superficial	27 (9.6%)
	Unknown	1 (0.3%)
Exposure on lower limb	Scratch	5 (1.8%)
	Bite	275 (98.2%)
Mucosa lesion	Yes	14 (1.4%)
	No	998 (98.6%)
Type of mucosa lesion	Deep	13 (92.9%)
	Superficial	1 (7.1%)
Exposure on mucosa	Scratch	2 (14.3%)
	Lick	1 (7.1%)
	Bite	11 (78.6%)
Number of lesions	Unknown	14 (1.4%)
	Multiple	624 (61.7%)
	Single	374 (37.0%)

Data presented as the number of individuals (N) and percentage (%).

Injuries on the trunk and mucosa did not present significant statistical differences regarding the different age groups. Trunk lesions were reported in 13 (5.3%), 26 (9.7%), and 56 (11.2%) children in the 0–3 years old, 4–6 years old, and 7–14 years old groups, respectively, while mucosa injuries were found in two (0.8%), seven (2.6%), and five (1%) children, respectively.

The lesions found were usually multiple (*n* = 624; 61.7%). In 374 (37%) cases, injuries were reported as single. Multiple lesions were the most prevalent in all age groups, occurring in 65.1% (*n* = 157), 59.2% (*n* = 157), and 63% (*n* = 310) children, respectively, in the 0–3 years old, 4–6 years old, and 7–14 years old groups (Table 2).

As for the type of exposure, regardless of the part of the body affected, bites were the most frequent, appearing in over 78% of the cases on the parts of the body already mentioned, followed by scratch and lick. Regarding the type of lesion, deep lesions were found in over 84% of the cases on different parts of the body. Tearing was reported in 29 (7.7%) cases on the head and neck, 14 (5.0%) cases on lower limbs, and seven (2.0%) cases on upper limbs, but was not reported on the children’s mucosa or trunk (Table 3).

This study did not find any death records.

## Discussion

Accidents involving dogs and children are quite frequent in our country and worldwide. In our region, the number of children assisted due to dog attack lesions annually is very high, which was also reported in studies developed at large health services in the United States of America, with an average of 120 cases per year, per institution (3, 5, 25, 26). Most accidents involved male children from 7 to 14 years old, occurred in an external environment and were not provoked by the victims. The injuries were mostly deep and multiple and mainly found on the head/neck region.

Our study showed that boys are more likely to be bitten by dogs, this fact had been previously reported (3, 5, 7, 12–15, 17, 18, 24, 25, 27–30). To the best of our knowledge, only one large study developed in the United States of America reported a higher number of girls involved in accidents with dogs (6).

Lack of standardization in the division of age groups in the studies under analysis hampered the comparison of data among them. In this case-by-case analysis, the predominance of accidents in the 7–14 years old group was observed. In a study by Bykowski et al., a prevalence of accidents with dogs in children under 5 years old was reported (3). According to the herein report, when joining the age groups, children under 6 years old were involved in the highest number of cases, which agrees with other studies (3, 6, 7, 12, 17, 25, 28, 31–33).

Children under 3 years old were twice as much more likely to be bitten by dogs in their own houses than those aged seven or over. When the age increased, we noticed an increase in the frequency of accidents with unknown dogs. This data also agrees with the literature (5, 6, 10, 12, 14, 23, 31, 32, 34).

In this study, the prevalence of accidents with non-domiciled or semi-domiciled dogs was high (68.2%). In a study carried out in Texas, United States of America, bites by street dogs were reported in 15.5% of cases only (22), while an European study reported bites in public places in 35% of the cases (34). This might indicate that in our country, the number of animals abandoned on the streets is quite high and that children might be unaccompanied by adult individuals at the time of the accident, as reported by Kahn et al. (34).

We observed that the accidental cause in dog attacks was the most frequent when each age group was investigated separately. McGuire et al. reported a similar finding in their study (7). However, we noticed that the number of provoked accidents increased when the children’s age decreased. The trend observed of younger children being mostly attacked at home and for a provoked cause might indicate some failure in the supervision of the child/dog interaction by the family members. The children’s familiarity with their pets might generate a false sensation of safety and result in a longer time of exposure in the absence of an adult individual, and risky behavior with dogs might be sometimes neglected by the parents (23, 31, 32, 34). Education actions targeting parents of children in this age group would result in greater benefit than those directed to the children.

Older children can also benefit from education actions suitable for their age group since, in general, they are more likely to experience lesions due to accidental causes in interactions in external environments with unknown dogs. This might indicate that street dogs might show a more aggressive and territorial behavior than pets, which would demand greater care when interacting with them, and the need for public policies to control these animals (23, 31).

Head and neck lesions were the most frequent, confirming findings of previous studies (5–7, 12, 14–17, 19, 20, 25, 28, 32–35). A higher frequency of these lesions in younger children was also found in other studies (3, 5, 9, 15, 17, 19, 28, 32, 34, 36–38). The incidence of lesions on these parts of the body has been ascribed to the fact that younger children are shorter, have a proportionally bigger head, crawl, and play on the floor, making this region more accessible to the dog (32, 33, 39). Other reasons implied are their exploratory attitude, lack of ability to recognize signs of a probable attack, and risky behavior such as kissing and hugging the animal (39).

When the age increases, the frequency of head and neck lesions decreases, and an increase in the frequency of bites on the limbs is observed. This finding was reported in other studies (3, 5, 9, 15, 17, 24, 25, 32, 33). Such progressive reduction is temporally correlated to the children’s neuropsychomotor development since when they grow, they tend to show better recognition of attack risk signs and become better able to protect themselves. However, they also start to take part in activities that increase the risk of bites occurring on the more peripheral regions of the body, such as upper and lower limbs, patting, feeding, and playing/running with the animals, for example.

Multiple and deep lesions were the most prevalent. In a study carried out in Austria, deep lesions were reported in 85% of the cases (19). McGuire et al. reported superficial lesions that did not affect muscles in over 90% of the cases in their study (7), while Zangari et al. reported this kind of lesions in 75.9% of the cases (12). However, the definition of the type of lesion was different in each study. Most of the lesions described as tearing were found on the head or neck of the children, according to the herein report. This finding has similar results in the referenced literature (12, 18, 29).

The dogs’ breed is described as one of the most important risk factors regarding accidents with humans, leading to the implementation of accident prevention regulations. However, the exact report of breeds did not occur in around 53 and 59% of the American cohorts in 1997 and from 2007 to 2011, respectively, which was also observed in other studies in over half of the cases (3, 17, 19, 24, 28, 32, 38). In this study, we also verified a high frequency of unreported breeds (50.6% of the cases). The dog’s breed is usually reported by the child or their family, which makes this parameter susceptible to errors of identification. This raises some doubt regarding the accuracy of the information related to the dogs’ breeds described in this study or previous studies on dog attacks. A study developed in Italy reported mongrel dogs as the most frequently breed involved in accidents with children (12), which agrees with our study. Among the reported breeds, Pit Bull was the most prevalent, which agrees with most of the literature (7, 14, 17, 22–25, 27, 28, 30, 32, 33, 35). Curiously, Poodle dogs were the third breed most cited, despite being a breed considered suitable for families with children and not highly reported in the literature about dog bites.

The data from the present study helps develop measures to prevent accidents with dogs. In the literature, there is no direct evidence that educational programs can reduce dog bite rates in children and adolescents. However, studies have shown that pre-school-aged children can learn to recognize dogs’ emotions and understand how it is safe to approach a dog (40–44). Some studies have been published demonstrating the positive effects of early training and socialization of dogs, and there is little argument against the potential of this training (45). However, to the best of our knowledge, no studies have demonstrated that these efforts lead to a decrease in pediatric dog bite rates. Thus, multifactor prevention programs are likely to be more efficient in reducing the number of such incidents. These programs ideally include guidance for parents of preschool children with strict rules for supervising contact between children and dogs, educational programs targeting older children and teenagers, dog training exercises, and dog behavior interpretation training for tutors (40, 46, 47). Sites like www.thebluedog.org can be a good resource for information on how people, especially children, should interact and approach dogs in specific situations (44, 48). So far, the evidence does not suggest that educational actions are effective as the only strategy to reduce dog bite injuries and their consequences (42). In this context, the development of public policies, with specific legislation for high-risk breeds and the search for control of the animal population abandoned on the streets, such as castration programs, support, and encouragement to rescue groups of street dogs, maintenance of shelters for dogs with rehabilitation programs and encouraging the adoption and conscious ownership of domestic animals, is of fundamental importance (40).

One of the limitations of this study resulted from the restructuring that occurred in our health service and in the São Paulo State service regarding the application of the anti-rabies serum, which had its production reduced worldwide from 2015 onward. This caused a change in the reporting and referral of dog attack cases, which led to a considerable reduction in the number of cases reported in 2019. It was difficult to access the medical records of 2018. Therefore, these years presented the lowest number of cases analyzed in this study. A selection bias might also be found in this study since only children with lesions considered serious enough by the parents, or other health professionals sought the emergency service for care. Undoubtedly, many dog bites occur that do not reach the emergency units of tertiary hospitals. Thus, the number of accidents reported in this study might not precisely represent the actual number of accidents with dogs that occurred in the community in the period evaluated.

This is one of the few studies that investigates differences between dog attacks in different age groups in pediatrics in Brazil. This study confirms previous findings regarding patients’ age and the distribution of lesions on the patients’ bodies, it also evidences that this type of accident remains a relevant public health issue in developing countries.

We concluded that dog attacks are more frequent in boys and that younger children have greater chances of being attacked inside their homes and by domiciled dogs, which results in a greater incidence of head and neck lesions in this age group. Older children tend to present more limb injuries caused by dogs that cannot be observed, which implies the need of anti-rabies agents, consequently, with higher costs to the health services.

## Data availability statement

The original contributions presented in this study are included in the article/Supplementary material, further inquiries can be directed to the corresponding author.

## Ethics statement

The studies involving human participants were reviewed and approved by the University of Campinas. Written informed consent to participate in this study was provided by the participants’ legal guardian/next of kin.

## Author contributions

MM: study conception, design, data collection, data interpretation, writing, and reviewing. FM and AP: study conception, design, data analysis, interpretation, writing, and reviewing. LM: data analysis, interpretation, writing, and reviewing. AF: study conception, design, critical review of the relevant content, and final approval. All authors contributed to the article and approved the submitted version.

## References

[1] Aprenda a Prevenir. *Aprenda a Prevenir Acidentes com Bebês – Criança Segura 2022 [Internet].* (2022). Available online at: https://criancasegura.org.br/aprenda-a-prevenir/previna-acidentes-com-bebes/ (accessed May 12, 2022).

[2] Hawkins RD Williams JM. Scottish society for the prevention of cruelty to animals Scottish S. Childhood attachment to pets: associations between pet attachment, attitudes to animals, compassion, and humane behaviour. *Int J Environ Res Public Health.* (2017) 14:490. 10.3390/ijerph14050490 28481256 PMC5451941

[3] Bykowski MR Shakir S Naran S Smith DM Goldstein JA Grunwaldt L Pediatric dog bite prevention: are we barking up the wrong tree or just not barking loud enough? *Pediatr Emerg Care.* (2017) 35:618–23. 10.1097/PEC.0000000000001132 28398940

[4] ABINPET. *Informações Gerais do Setor Pet 2022 [Internet].* (2022). Available online at: http://abinpet.org.br/infos_gerais/ (accessed May 12, 2022).

[5] Golinko MS Arslanian B Williams JK. Characteristics of 1616 consecutive dog bite injuries at a single institution. *Clin Pediatr (Phila).* (2017) 56:316–25. 10.1177/0009922816657153 27400935

[6] Fein J Bogumil D Upperman JS Burke RV. Pediatric dog bites: a population-based profile. *Inj Prev.* (2018) 25:290–4. 10.1136/injuryprev-2017-042621 29439149

[7] McGuire C Morzycki A Simpson A Williams J Bezuhly M. Dog bites in children: a descriptive analysis. *Plastic Surg (Oakville, Ont).* (2018) 26:256–62. 10.1177/2292550318767924 30450344 PMC6236502

[8] Beck AM Jones BA. Unreported dog bites in children. *Public Health Rep.* (1985) 100:315. PMC1424765 3923540

[9] Cook JA Sasor SE Soleimani T Chu MW Tholpady SS. An epidemiological analysis of pediatric dog bite injuries over a decade. *J Surg Res.* (2020) 246:231–5. 10.1016/j.jss.2019.09.013 31606513

[10] Basco AN McCormack ER Basco WT. Age– and sex-related differences in nonfatal dog bite injuries among persons aged 0-19 treated in hospital emergency departments, United States, 2001-2017. *Public health rep.* (2020) 135:238–44. 10.1177/0033354920904072 32040928 PMC7036613

[11] Náhlík J Eretová P Chaloupkóvá H Vostrá-Vydrová H Fiala Šebková N Trávníček J. How parents perceive the potential risk of a child-dog interaction. *Int J Environ Res Public Health.* (2022) 19:564. 10.3390/ijerph19010564 35010826 PMC8744742

[12] Zangari A Cerigioni E Nino F Guidi R Giulia C Piergentili R Dog bite injuries in a tertiary care children’s hospital: a seven-year review. *Pediatr Int.* (2021) 63:575–80. 10.1111/ped.14484 32979010

[13] Tulloch JSP Minford S Pimblett V Rotheram M Christley RM Wesgarth C. Paediatric emergency department dog bite attendance during the COVID-19 pandemic: an audit at a tertiary children’s hospital. *BMJ Paediatr Open.* (2021) 5:e0001040. 10.1136/bmjpo-2021-001040 33884313 PMC8023759

[14] Reuter Muñoz KD Powell LE Andersen ES Nye AD Powers JM Rhodes J Analysis of pediatric dog bite injuries at a level 1 trauma center over 10 years. *Ann Plast Surg.* (2021) 86:S510–6. 10.1097/SAP.0000000000002928 34100808

[15] McLoughlin RJ Cournoyer L Hirsh MP Cleary MA Aidlen JT. Hospitalizations for pediatric dog bite injuries in the United States. *J Pediatr Surg.* (2020) 55:1228–33. 10.1016/j.jpedsurg.2019.06.025 31326111

[16] Sulaiman A Liang D Gianoutsos M Moradi P. Paediatric dog bite injuries: a 10-year retrospective cohort analysis from Sydney children’s hospital. *ANZ J Surg.* (2022) 92:1149–52. 10.1111/ans.17581 35229428

[17] Ramgopal S Brungo LB Bykowski MR Pitetti RD Hickey RW. Dog bites in a U.S. County: age, body part and breed in paediatric dog bites. *Acta Paediatr.* (2018) 107:893–9. 10.1111/apa.14218 29331048 PMC8278816

[18] Tam B Matsushima K Chiba H Park T Slocum C Lam L Nationwide analysis of dog bite injuries: different age groups, Different Injury Patterns. *Am Surg.* (2021) 87:1612–5. 10.1177/00031348211024657 34130512

[19] Schalamon J Ainoedhofer H Singer G Petnehazy T Mayr J Kiss K Analysis of dog bites in children who are younger than 17 years. *Pediatrics.* (2006) 117:e374–9. 10.1542/peds.2005-1451 16510617

[20] Lang ME Klassen T. Dog bites in Canadian children: a five-year review of severity and emergency department management. *CJEM.* (2005) 7:309–14. 10.1017/s1481803500014494 17355691

[21] Peixoto AO Sarti L Uzun RS Belluomini F Takesaki NA Marson FAL Bite of *Canis lupus* familiaris in an infant causing skull injury and neurological sequelae. *J Vet Behave.* (2020) 38:103–6. 10.1016/j.jveb.2020.03.006

[22] Hasoon BC Shipp AE Hasoon J. A look at the incidence and risk factors for dog bites in unincorporated Harris County, Texas, USA. *Vet World.* (2020) 13:419–25. 10.14202/vetworld.2020.419-425 32367944 PMC7183464

[23] Reisner IR Nance ML Zeller JS Houseknecht EM Kassam-Adams N Wiebe DJ. Behavioural characteristics associated with dog bites to children presenting to an urban trauma centre. *Inj Prev.* (2011) 17:348–53. 10.1136/ip.2010.029868 21444335

[24] Kaye AE Belz JM Kirschner RE. Pediatric dog bite injuries: a 5-year review of the experience at the children’s hospital of Philadelphia. *Plast Reconstr Surg.* (2009) 124:551–8. 10.1097/PRS.0b013e3181addad9 19644273

[25] Bernardo LM Gardner MJ Rosenfield RL Cohen B Pitetti R. A comparison of dog bite injuries in younger and older children treated in a pediatric emergency department. *Pediatr Emerg Care.* (2002) 18:247–9. 10.1097/00006565-200206000-00024 12066018

[26] Morales C Falcón N Hernández H Férnandez C. Accidentes por mordedura canina, casos registrados en un hospital de niños de Lima, Perú 1995-2009. *Rev Peru Med Exp Salud Publica.* (2011) 28:639–42. 10.1590/S1726-46342011000400011 22241261

[27] Tuckel PS Milczarski W. The changing epidemiology of dog bite injuries in the United States, 2005–2018. *Inj Epidemiol.* (2020) 7:57. 10.1186/s40621-020-00281-y 33129353 PMC7603431

[28] Daniels DM Ritzi RB O’Neil J Scherer LR. Analysis of nonfatal dog bites in children. *J Trauma.* (2009) 66(Suppl. 3):S17–22. 10.1097/TA.0b013e3181937925 19276721

[29] Bjork A Holman RC Callinan LS Hennessy TW Cheek JE McQuiston JH. Dog bite injuries among American Indian and Alaska native children. *J Pediatr.* (2013) 162:1270–5. 10.1016/j.jpeds.2012.11.087 23332462

[30] Garvey EM Twitchell DK Ragar R Egan JC Jamshidi R. Morbidity of pediatric dog bites: a case series at a level one pediatric trauma center. *J Pediatr Surg.* (2015) 50:343–6. 10.1016/j.jpedsurg.2014.09.051 25638634

[31] Matthias J Templin M Jordan MM Stanek D. Cause, setting and ownership analysis of dog bites in Bay County, Florida from 2009 to 2010. *Zoonoses Public Health.* (2015) 62:38–43. 10.1111/zph.12115 24712701

[32] Chiam SC Solanki NS Lodge M Higgins M Sparnon AL. Retrospective review of dog bite injuries in children presenting to a South Australian tertiary children’s hospital emergency department. *J Paediatr Child Health.* (2014) 50:791–4. 10.1111/jpc.12642 25041425

[33] Abraham JT Czerwinski M. Pediatric dog bite injuries in central Texas. *J Pediatric Surg.* (2019) 54:1416–20. 10.1016/j.jpedsurg.2018.09.022 30473254

[34] Kahn A Bauche P Lamourex J. Child victims of dog bites treated in emergency departments: a prospective survey. *Eur J Pediatr.* (2003) 162:254–8. 10.1007/s00431-002-1130-6 12647199

[35] Alizadeh K Shayesteh A Xu ML. An algorithmic approach to operative management of complex pediatric dog bites: 3-year review of a level I regional referral pediatric trauma hospital. *Plast Reconstr Surg Glob Open.* (2017) 5:e1431. 10.1097/GOX.0000000000001431 29184724 PMC5682160

[36] Bashir K Haq I Khan SMS Qurieshi MA. One-year descriptive analysis of patients treated at an anti-rabies clinic-A retrospective study from Kashmir. *PLoS Negl Trop Dis.* (2020) 14:e0007477. 10.1371/journal.pntd.0007477 32841227 PMC7473535

[37] Nkomo M Mahomed Z Laher AE. An audit of patients with dog-bite wounds presenting to a tertiary level hospital emergency department in South Africa. *Cureus.* (2020) 12:e6558. 10.7759/cureus.6558 32042530 PMC6996530

[38] Hon KL Fu CC Chor CM Tang PS Leung TF Man CY Issues associated with dog bite injuries in children and adolescents assessed at the emergency department. *Pediatr Emerg Care.* (2007) 23:445–9. 10.1097/01.pec.0000280509.67795.a9 17666924

[39] Rajshekar M Blizzard L Julian R Williams AM Tennant M Forrest A The incidence of public sector hospitalisations due to dog bites in Australia 2001-2013. *Aust N Z J Public Health.* (2017) 41:377–80. 10.1111/1753-6405.12630 28712151

[40] Kakeman M Oxley JA Owczarczak-Garstecka SC Westgarth C. Pet dog bites in children: management and prevention. *BMJ Paediatr Open.* (2020) 4:e000726. 10.1136/bmjpo-2020-000726 32821860 PMC7422634

[41] Chapman S Cornwall J Righetti J Sung L. Preventing dog bites in children: randomised controlled trial of an educational intervention. *BMJ.* (2000) 320:1512–3. 10.1136/bmj.320.7248.1512 10834894 PMC27395

[42] Duperrex O Blackhall K Burri M Jeannot E. Education of children and adolescents for the prevention of dog bite injuries. *Cochrane Database Syst Rev.* (2009) 15:CD004726. 10.1002/14651858.CD004726.pub2 PMC11738851 19370606

[43] Shen J Rouse J Godbole M Wells HL Shilpa B Schwebel DC. Systematic review: interventions to educate children about dog safety and prevent pediatric dog-bite injuries: a meta-analytic review. *J Pediatr Psychol.* (2017) 42:779–91. 10.1093/jpepsy/jsv164 26773009 PMC5896610

[44] Meints K de Keuster T. Brief report: don’t kiss a sleeping dog: the first assessment of “the blue dog” bite prevention program. *J Pediatr Psychol.* (2009) 34:1084–90. 10.1093/jpepsy/jsp053 19578138

[45] Hsu Y Serpell JA. Development and validation of a questionnaire for measuring behavior and temperament traits in pet dogs. *J Am Vet Med Assoc.* (2003) 223:1293–300. 10.2460/javma.2003.223.1293 14621216

[46] Schwebel DC Morrongiello BA Davis AL Stewart J Bell M. The Blue Dog: evaluation of an interactive software program to teach young children how to interact safely with dogs. *J Pediatr Psychol.* (2012) 37:272–81. 10.1093/jpepsy/jsr102 22173883

[47] Shields WC McDonald EM Stepnitz R McKenzie LT Gielen AC. Dog bites: an opportunity for parent education in the pediatric emergency department. *Pediatr Emerg Care.* (2012) 28:966–70. 10.1097/PEC.0b013e31826c6c13 23023457 PMC3674576

[48] The Blue Dog. *Safe Relationships Between Children and Dogs – The Blue Dog 2022 [Internet].* (2022). Available online at: https://www.thebluedog.org/en/ (accessed July 6, 2022).

