# Supplementary material for: Epidemiological profile of dog attacks to patients under 14 years old assisted at the pediatric referral emergency unit of a tertiary hospital in Campinas, Brazil
Source: Front Pediatr. 2022 Aug 3;10:963803. doi: 10.3389/fped.2022.963803 (PMC9382190; doi:10.3389/fped.2022.963803)
Supplement: Supplementary file 1 [file Data_Sheet_1.docx]

**Supplementary Material**

**Title:** Epidemiological profile of dog attacks to patients under 14 years old assisted at the Pediatric referral emergency unit of a tertiary hospital in Campinas, Brazil

**Short title:** Dog attacks in pediatrics

| **Supplementary Table 1.** Number of annual cases of dog bite in individuals under 14 years old assisted at the Pediatric referral emergency unit of a tertiary hospital in Campinas, Brazil. | |
| --- | --- |
| **Year of inclusion** | **N (%)** |
| 2010 | 122 (12.1%) |
| 2011 | 121 (12.0%) |
| 2012 | 163 (16.1%) |
| 2013 | 127 (12.5%) |
| 2014 | 96 (9.5%) |
| 2015 | 92 (9.1%) |
| 2016 | 92 (9.1%) |
| 2017 | 107 (10.6%) |
| 2018 | 26 (2.6%) |
| 2019 | 66 (6.5%) |

Data presented as number of individuals (N) and percentage (%)

| **Supplementary Table 2.** Number of cases of dog bites in individuals under 14 years old assisted at the Pediatric referral emergency unit of a tertiary hospital in Campinas, Brazil, according to the municipality where the accident occurred. | | | |
| --- | --- | --- | --- |
| **City** | **Accident** | **City** | **Accident** |
| Alterosa* | 1 (0.1%) | Limeira | 1 (0.1%) |
| Americana | 44 (4.3%) | Louveira | 2 (0.2%) |
| Amparo | 1 (0.1%) | Mogi Guaçu | 2 (0.2%) |
| Aparecida do Norte | 1 (0.1%) | Monguaguá | 1 (0.1%) |
| Araraquara | 1 (0.1%) | Monte Mor | 10 (1.0%) |
| Arthur Nogueira | 5 (0.5%) | Monte Sião* | 1 (0.1%) |
| Atibaia | 4 (0.4%) | Nazaré Paulista | 1 (0.1%) |
| Bom Jesus dos Perdões | 2 (0.2%) | Nova Odessa | 16 (1.6%) |
| Botucatu | 1 (0.1%) | Paulinia | 37 (3.7%) |
| Bragança Paulista | 2 (0.2%) | Pedreira | 8 (0.8%) |
| Campinas | 438 (43.3%) | Piracaia | 1 (0.1%) |
| Campo Limpo Paulista | 1 (0.1%) | Piracicaba | 2 (0.2%) |
| Conchal | 6 (0.6%) | Poá | 1 (0.1%) |
| Cosmópolis | 19 (1.9%) | Pouso Alegre* | 1 (0.1%) |
| Francisco Beltrão** | 1 (0.1%) | Praia Grande | 1 (0.1%) |
| Guarujá | 3 (0.3%) | Salto | 1 (0.1%) |
| Guarulhos | 1 (0.1%) | Santa Cruz das Palmeiras | 1 (0.1%) |
| Holambra | 2 (0.2%) | Santo Antônio de Posse | 11 (1.1%) |
| Hortolândia | 98 (9.7%) | Santos | 1 (0.1%) |
| Ignorado | 25 (2.5%) | São João da Boa Vista | 2 (0.2%) |
| Indaiatuba | 32 (3.2%) | São Paulo | 3 (0.3%) |
| Itaobim | 1 (0.1%) | São Pedro | 1 (0.1%) |
| Itatiba | 8 (0.8%) | Sorocaba | 1 (0.1%) |
| Itupeva | 3 (0.3%) | Santa Gertrudes | 1 (0.1%) |
| Jacutinga* | 1 (0.1%) | Santa Maria da Serra | 1 (0.1%) |
| Jaguariúna | 23 (2.3%) | Sumaré | 96 (9.5%) |
| Jales | 1 (0.1%) | Ubatuba | 1 (0.1%) |
| Jarinú | 4 (0.4%) | Valinhos | 20 (2.0%) |
| Joanopolis | 1 (0.1%) | Várzea Paulista | 2 (0.2%) |
| Jundiaí | 11 (1.1%) |  |  |
| **Total** | 1,012 |  |  |

*, Minas Gerais; **, Paraná. Data presented as number of individuals (N) and percentage (%).

| **Supplementary Table 3.** Breeds of dogs that attacked individuals under 14 years old assisted at the Pediatric referral emergency unit of a tertiary hospital in Campinas, Brazil. | |
| --- | --- |
| **Breed** | **N (%)** |
| Akita | 3 (0.3%) |
| Basset Hound | 11 (1.1%) |
| Beagle | 1 (0.1%) |
| Border Collie | 1 (0.1%) |
| Boxer | 1 (0.1%) |
| Bull Terrier | 1 (0.1%) |
| Bulldog | 1 (0.1%) |
| Chow chow | 6 (0.6%) |
| Cocker Spaniel | 6 (0.6%) |
| Dalmatian | 3 (0.3%) |
| Doberman | 1 (0.1%) |
| *Fila Brasileiro* | 2 (0.2%) |
| *Fox Paulistinha* | 2 (0.2%) |
| Golden Retriever | 1 (0.1%) |
| Unknown | 512 (50.6%) |
| Labrador | 6 (0.6%) |
| Lhasa Apso | 1 (0.1%) |
| German Shepherd | 14 (1.4%) |
| Belgium Malinois | 2 (0.2%) |
| Pinscher | 7 (0.7%) |
| Pit Bull | 36 (3.6%) |
| Poodle | 13 (1.3%) |
| Rottweiler | 10 (1.0%) |
| Schnauzer | 1 (0.1%) |
| Shih Tzu | 2 (0.2%) |
| Mongrel (mixed-breed) | 366 (36.2%) |
| Yorkshire | 2 (0.2%) |
| Total | 1,012 |

Data presented as number of individuals (N) and percentage (%).
